# Additions to the Genus *Helicosporium* (Tubeufiaceae, Tubeufiales) from China with an Identification Key for *Helicosporium* Taxa

**DOI:** 10.3390/jof9070775

**Published:** 2023-07-22

**Authors:** Xing-Juan Xiao, Jian Ma, Li-Juan Zhang, Ning-Guo Liu, Yuan-Pin Xiao, Xing-Guo Tian, Zong-Long Luo, Yong-Zhong Lu

**Affiliations:** 1 School of Food and Pharmaceutical Engineering, Guizhou Institute of Technology, Guiyang 550003, China; juanj0826@163.com (X.-J.X.); yanmajian@163.com (J.M.); 6471105010@lamduan.mfu.ac.th (L.-J.Z.); liuningguo11@gmail.com (N.-G.L.); emmaypx@gmail.com (Y.-P.X.); xgtian2023@sina.com (X.-G.T.); 2 Center of Excellence in Fungal Research, Mae Fah Luang University, Chiang Rai 57100, Thailand; 3 College of Agriculture and Biological Science, Dali University, Dali 671003, China; luozonglongfungi@163.com

**Keywords:** six new taxa, ascomycetes, asexual morph, filamentous fungi, phylogeny, taxonomy

## Abstract

Helicosporous hyphomycetes is a group of filamentous fungi that shows promising application prospects in metabolizing bioactive natural compounds. During a study of helicosporous fungi in China, six new helicosporous taxa were collected and isolated from decaying wood in Guangxi Zhuang Autonomous Region, China. Morphological comparisons with multi-gene phylogenetic analyses revealed that these six taxa belong to *Helicosporium* (Tubeufiaceae, Tubeufiales), and they were recognized as three novel species and were named *Helicosporium liuzhouense, H. multidentatum,* and *H. nanningense*. Detailed descriptions and illustrations of the newly discovered taxa and comparisons with similar fungi are provided. In addition, a list and a key to accepted *Helicosporium* species are provided.

## 1. Introduction

Based on the type species *H. vegetum*, Nees [1] established *Helicosporium* as one of the earliest described genera of helicosporous hyphomycetes. The majority of this group’s species inhabit subtropical to tropical habitats [2,3,4,5,6,7,8,9,10,11]. Typically, they inhabit woody substrates in terrestrial and freshwater environments [3,7,8,9,10,12]. Index Fungorum (accessed on 20 May 2023) [13] currently lists 105 taxa of *Helicosporium*, of which 75 species have been excluded or transferred to other genera. Most of these taxa were transferred to the genera *Helicoma* Corda and *Neohelicosporium* Y.Z. Lu, J.C. Kang & K.D. Hyde. Currently, there are eighteen accepted species of *Helicosporium*, and twelve of which have molecular data [3,7,8,10,11].

All species in this genus are reported to have a helicosporous asexual morph. There are three species with reported sexual morph, *viz., H. flavum* Brahaman., Y.Z. Lu, Boonmee & K.D. Hyde, *H. sexuale* Boonmee, Promputtha & K.D. Hyde, and *H. vegetum* [7,8,14]. The sexual morph of *Helicosporium* is characterized by solitary, yellowish brown, globose to subglobose ascomata, cylindric-clavate, eight-spored bitunicate asci, and hyaline to yellowish brown, fusiform ascospores [7,8,14]. The asexual morph is distinguished by pale yellow to yellow green colonies on the natural woody substratum, erect, setiferous, cylindrical conidiophores with denticulate conidiogenous cells arising laterally from the lower portions of conidiophores resembling tiny tooth-like or bladder-like protrusions, and hyaline to yellow green, pleurogenous helicoid conidia that are smaller than 25 μm diameter with conidial filaments usually not exceeding 4 μm thickness [10,11].

*Helicosporium* fungi have the potential of producing bioactive secondary metabolites. The antimicrobial activity of *Helicosporium* was first reported by Hardy and Sivasithamparam [15]. The main antimicrobial constituent, 2-methyl resorcinol, was isolated from *Helicosporium* sp. KCTC 0635BP by Choi et al. [16]. It was reported to have cytotoxicity against mammalian cells and antimicrobial activity against various types of fungi and bacteria [16].

During a study of helicosporous hyphomycetes in China, six new helicosporous taxa were collected from Guangxi Zhuang Autonomous Region. Three new species, *Helicosporium liuzhouense*, *H. multidentatum, and H. nanningense*, were identified based on morphological evidence and phylogenetic analyses of combined LSU, ITS, *tef1α,* and *rpb2* sequence data. The results of the PHI test support the taxonomic classification of these three newly discovered species. The present study provides descriptive and illustrative morphological information as well.

## 2. Materials and Methods

### 2.1. Sample Collection, Specimen Examination, and Isolation

**Figure 1:**
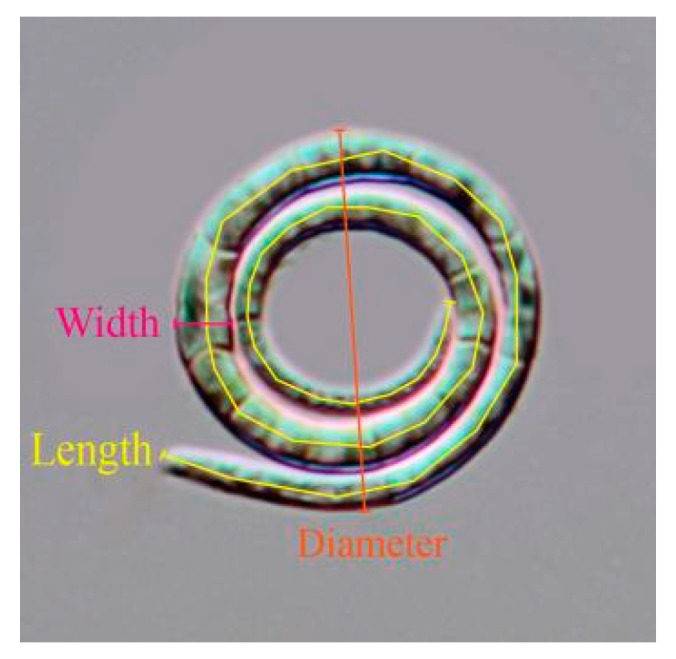
The measurement method for helicoid conidia.

Specimens of decaying wood were randomly sampled from terrestrial and freshwater habitats in Guangxi Zhuang Autonomous Region, China. Freshwater samples were incubated in sterile, moist plastic containers at room temperature for approximately 14 days. After two weeks of collection, fresh specimens were examined and observed using a stereomicroscope (SMZ 745 and SMZ 800N, Tokyo, Nikon, Japan). Morphological characteristics of fresh fungi specimens were recorded with stereomicroscopes fitted with a digital camera. The measurement data for the helicoid conidia includes diameter, thickness, and length. The specific measurement method is shown in Figure 1.

Single spore isolation was referred from the method described by Chomnunti et al. [17]. Purified cultures were cultured in a 25 °C incubator. The morphological features of colonies, including color, shape, and colony diameter, were recorded regularly.

The dried specimens were deposited in the Herbarium of Cryptogams Kunming Institute of Botany, Academia Sinica (KUN-HKAS), Kunming, China, and the Herbarium of Guizhou Academy of Agriculture Sciences (GZAAS), Guiyang, China. The cultures were deposited in the China General Microbiological Culture Collection Center (CGMCC), Beijing, China, and the Guizhou Culture Collection (GZCC), Guizhou, China.

### 2.2. DNA Extraction, PCR Amplification, and Sequencing

Using sterile toothpicks, 60-day-old mycelium was scraped from PDA plates and transferred to a 1.5-mL microcentrifuge tube. Using the Ezup fungus genomic DNA extraction kit (Sangon Biotech, Shanghai, China), DNA was extracted and sequenced following the manufacturer’s instructions. After obtaining the DNA of the fungal strains, EF1-983F/EF1-2218R, FRPB2-5F/FRPB2-7CR, ITS5/ITS4, and LR0R/LR5 were used as primers for amplification [18,19,20]. The amplification reactions of ITS, LSU, *tef1α*, and *rpb2* were carried out according to the methods of Lu et al. [21,22]. After PCR amplification, the products were analyzed using 1% agarose gel electrophoresis. The purification and sequencing of PCR products were completed by Beijing Tsingke Biological Engineering Technology and Services Co., Ltd. (Beijing, China).

### 2.3. Phylogenetic Analyses

BioEdit version 7.0.5.3 was used to inspect the original sequences. The forward and reverse sequences were assembled using SeqMan v. 7.0.0 (DNASTAR, Madison, WI, USA) software and submitted to the GenBank database. Based on recent publications, additional sequences similar to *Helicosporium* were downloaded from GenBank [7,8,9,10,11,14]. Sequence alignments for each locus were carried using the online multiple alignment program MAFFT version 7, and the alignments were further automatically adjusted using the trimAl tool [23]. The phylogenetic tree was constructed using the methods described by Ma et al. [24], which included Maximum Likelihood (ML) and Bayesian Inference (BI).

The phylogenetic trees were edited using FigTree v1.4.0 software. The edited trees and figure layouts were edited using Adobe PhotoShop CC 2018 and Adobe Illustrator CC 2021 (Adobe Systems, San Jose, CA, USA) software. Sequences generated in this study were uploaded to GenBank (Table 1).

### 2.4. Genealogical Concordance Phylogenetic Species Recognition (GCPSR) Analysis

Three new species, *H. liuzhouense*, *H. multidentatum,* and *H. nanningense,* were analyzed using GCPSR with closely related taxa from combined LSU-ITS*-tef1*-*α*-*rpb2* gene regions. The pairwise homoplasy index (PHI) test was carried out in SplitsTree4 [25,26]. It indicates that there is no statistically significant evidence for recombination for the selected taxa when the P-value is above 0.05. Both the LogDet transformation and splits decomposition options were used to reveal the relationship among closely related species.

## 3. Phylogenetic Results

The partial LSU-ITS-*tef1α*-*rpb2* nucleotide sequences were used to determine the phylogenetic position of our newly isolated taxa. The concatenated sequence matrix consisted of LSU (1–842 bp), ITS (843–1398 bp), *tef1α* (1399–2310 bp), and *rpb2* (2311–3337 bp), totaling 3337 characters for 30 taxa and two outgroups, *Acanthostigma chiangmaiense* (MFLUCC 10–0125) and *A. perpusillum* (UAMH 7237). The ML and BI analyses of the concatenated LSU-ITS-*tef1α*-*rpb2* dataset yielded similar tree topologies, and the ML tree is shown in Figure 1. The bootstrap support values of ML equal to or greater than 75%, and PP equal to or greater than 0.95 are given near the nodes as ML/PP, respectively.

**Figure 2:**
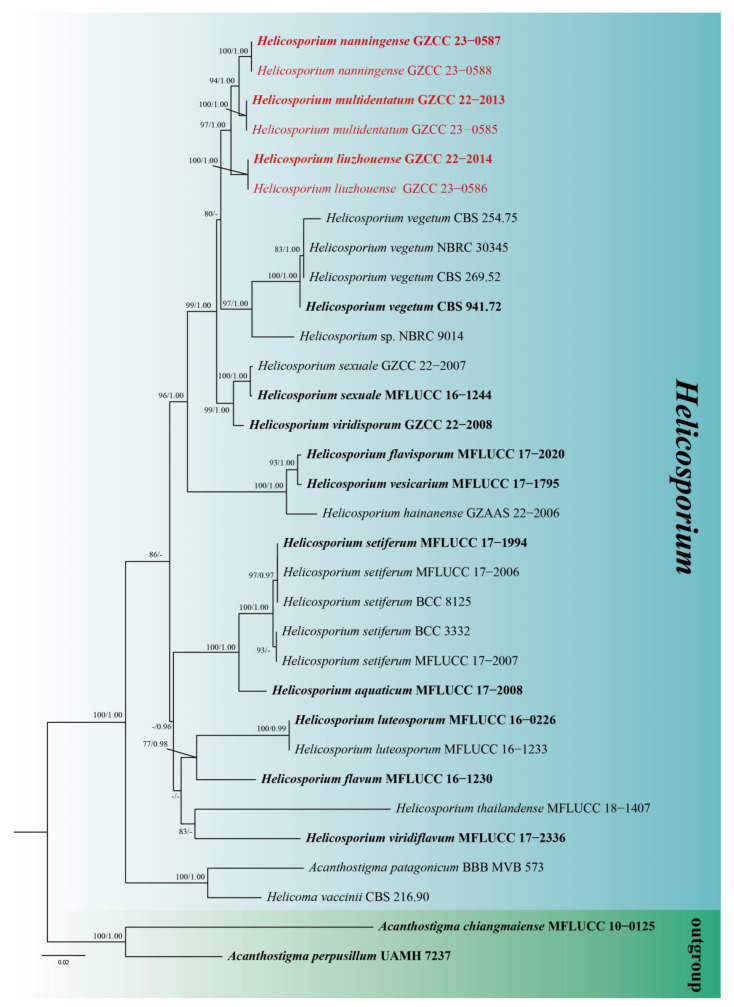
The phylogenetic tree generated using Maximum Likelihood (ML) analysis based on combined LSU, ITS, *tef1α*, and *rpb2* sequence data. ML and Bayesian Posterior Probabilities (PP) near the nodes are indicated as ML/PP. The *Acanthostigma chiangmaiense* (MFLUCC 10–0125) and *A*. *perpusillum* (UAMH 7237) were used as outgroup taxa. Ex-type strains are represented in bold. Newly generated sequences are represented in red.

The resulting multigene phylogenetic tree confirms that our newly isolated *Helicosporium liuzhouense*, *H. multidentatum,* and *H. nanningense* form a distinct clade from other taxa within the genus *Helicosporium* (Figure 2).

## 4. Genealogical Concordance Phylogenetic Species Recognition (GCPSR) Analysis

**Figure 3:**
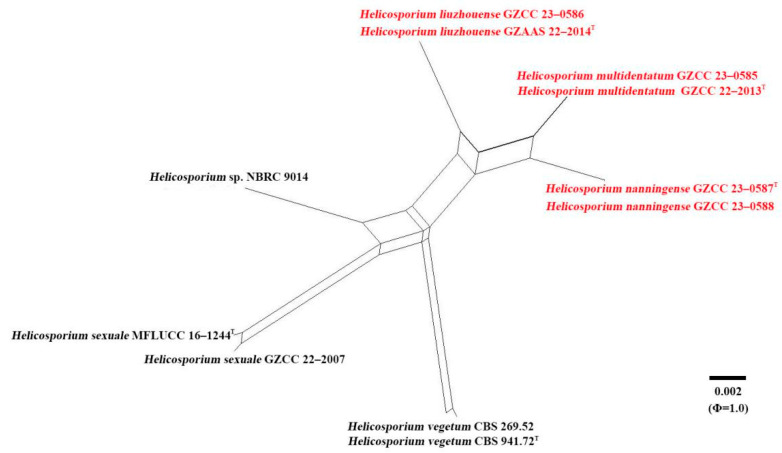
Results of the PHI test of *Helicosporium liuzhouense*, *H. multidentatum,* and *H. nanningense* with closely related species (combined LSU-ITS-*tef1α*-*rpb2*) using both LogDet transformation and splits decomposition. PHI test results (Φ_w_) < 0.05 indicate significant recombination within the dataset. New species are indicated in red, and type strains are marked with “^T^”.

Application of the PHI test to the concatenated tree-locus sequences of LSU-ITS-*tef1α*-*rpb2* revealed the recombination level within phylogenetically related species. No significant recombination events were observed between our species *Helicosporium liuzhouense*, *H. multidentatum,* and *H. nanningense* and closely related species in *Helicosporium* (Figure 3). The test results show Φ_w_ = 1 for the combined sequence data, Φ_w_ = 1 for LSU dataset, Φ_w_ = 0.80 for ITS dataset, Φ_w_ = 0.09 for *tef1α,* and Φ_w_ = 0.93 for *rpb2* data.

## 5. Taxonomy

**Figure 4:**
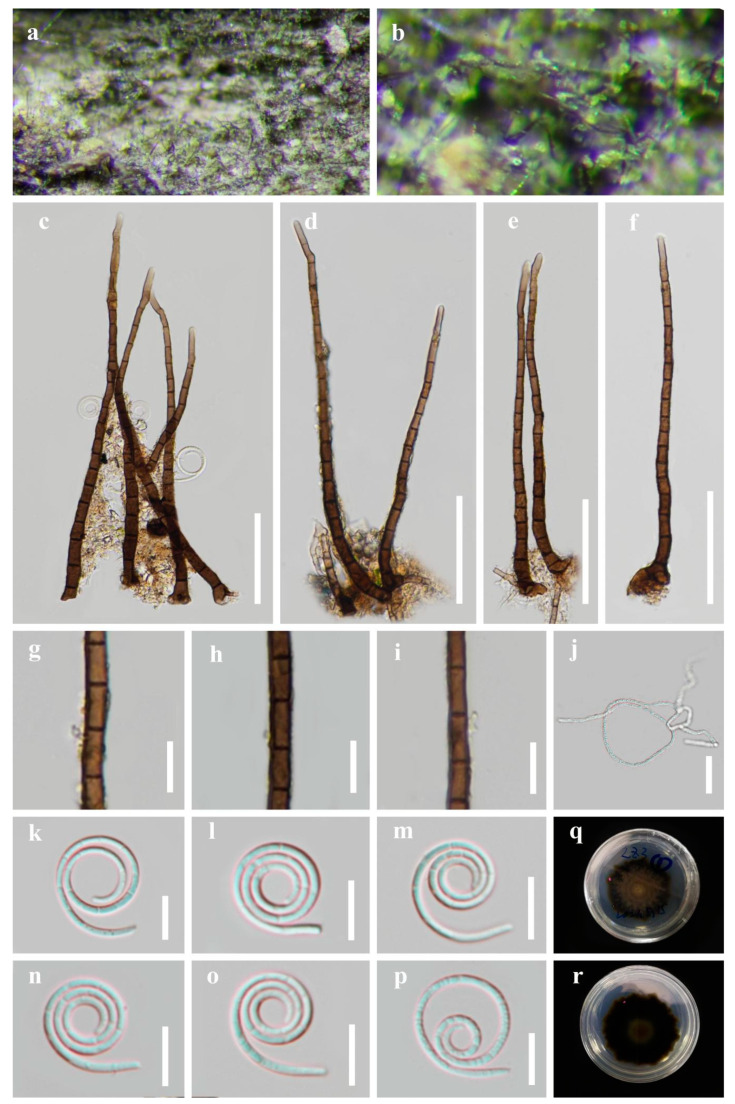
*Helicosporium liuzhouense* (HKAS 125865, holotype). (**a**,**b**) Colonies on natural substrate. (**c**–**f**) Conidiophores. (**g**–**i**) Conidiogenous cells. (**j**) Germinating conidium. (**k**–**p**) Conidium. (**q**–**r**) Anverse and reverse colonies on PDA. Scale bars: (**c**–**f**) 50 μm, (**j**) 20 μm, and (**g**–**i**,**k**–**p**) 10 μm.

*Helicosporium liuzhouense* X.J. Xiao, J. Ma & Y.Z. Lu, sp. nov., Figure 4.

Index Fungorum number: IF900461

Etymology: The epithet “*liuzhouense*” named after the city in which the holotype was found.

Holotype: HKAS 125865

*Saprobic* on decaying wood in a freshwater stream. *Sexual morph*: Unknown. *Asexual morph*: Hyphomycetous, helicosporous. *Colonies* on the substratum superficial, effuse, gregarious, bright lime green. *Mycelium* partly immersed, partly superficial, composed of branched, septate, hyphae, pale brown to brown hyphae. *Conidiophores* macronematous, mononematous, erect, flexuous or straight, unbranched, septate, apical sterile, cylindrical, (102) 110–180 (213) × 4–5 μm ($\bar{x}\;$= 145 × 5 μm, n = 25), brown to dark brown, thick-walled, smooth-walled. *Conidiogenous cells* holoblastic, monoblastic to polyblastic, determinate, cylindrical, with denticles, arising laterally from the lower portion of the conidiophores as tiny tooth-like protrusions, hyaline to pale brown, smooth-walled. *Conidia* solitary, pleurogenous, helicoid, rounded at tip, 13–15 μm diam., and conidial filament 1–2 μm wide ($\bar{x}$ = 14 × 1.5 μm, n = 25), 90–105 μm long, tightly coiled 2–3 times, becoming loosely coiled in water, indistinctly multi-septate, guttulate, hyaline to pale green, smooth-walled.

Culture characteristics: Conidia germinated on water agar and produced germ tubes within 8 h. The colonies grew on PDA, had a circular shape with a flat surface and undulate edge. They reached a size of 46 mm in 6 weeks at 25 °C and exhibited a pale brown center with brown edges on PDA.

Material examined: CHINA, Guangxi Zhuang Autonomous Region, Liuzhou City, Luzhai County, on decaying wood in a freshwater stream, 4 May 2021, Xing-Juan Xiao & Jian Ma, LZ3 (HKAS 125865, holotype; GZAAS 22–2014, isotype), ex-type living culture CGMCC, GZCC 22–2014. *Ibid*., LZ3-2 (HKAS 125870, paratype), living culture GZCC 23–0586.

Notes: Phylogenetically, *Helicosporium liuzhouense* is strongly supported as a sister species to *H. multidentatum* and *H. nanningense* (97% ML/1.00 PP). Morphologically, *Helicosporium liuzhouense* is distinguished from *H. multidentatum* by having conidiogenous cells with tiny tooth-like protrusions, while *H. multidentatum* has conidiogenous cells with integrated multi-dentate protrusions. Additionally, *H. liuzhouense* is characterized by shorter conidia (90–105 µm vs. 105–128 µm) and a larger conidial diameter (13–15 µm vs. 12–13 µm) compared to *H. multidentatum*. *Helicosporium liuzhouense* can be differentiated from *H. nanningense* by having shorter conidiophores (90–115 µm vs. 100–215 µm) and conidial filaments (82–92 µm vs. 90–105 µm). The PHI test provides strong evidence showing that they are separate species (Figure 3). Although *H. liuzhouense* and *H. viridisporum* Y.Z. Lu & J.C. Kang share similar conidiophores, conidiogenous cells, and conidial features [11], the phylogenetic analyses indicate that they are distinct species.

**Figure 5:**
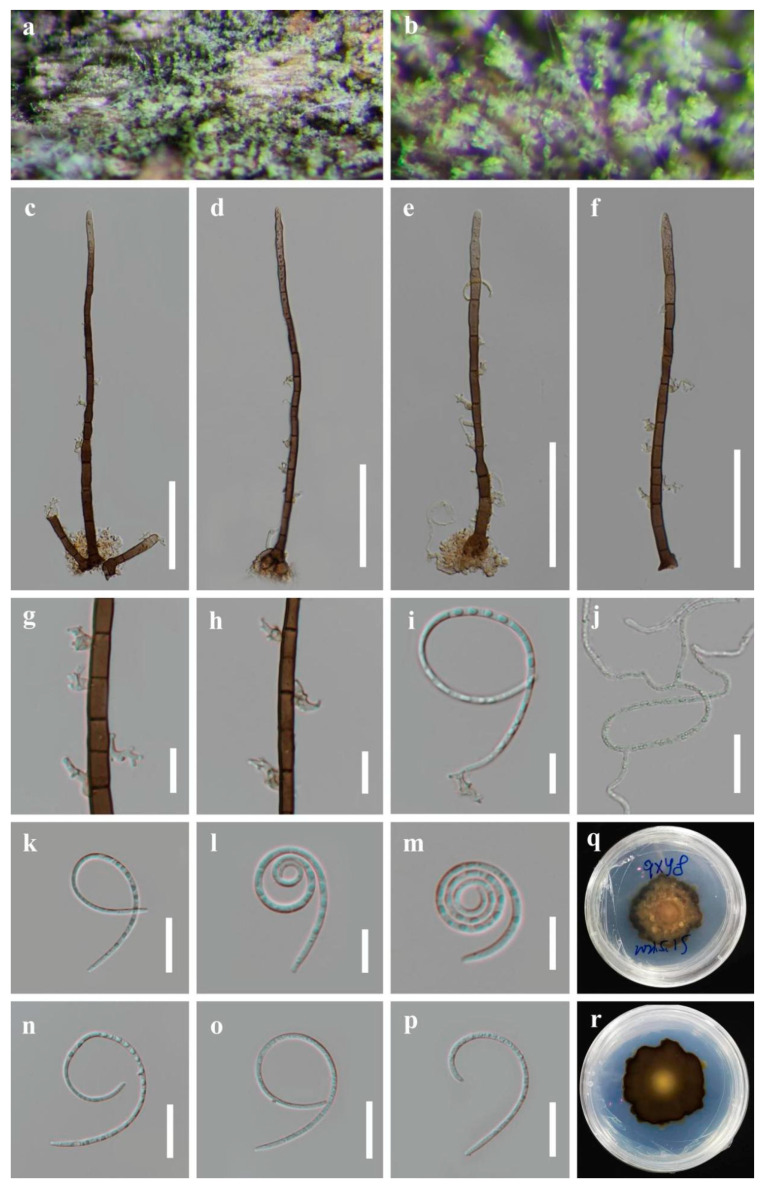
*Helicosporium multidentatum* (HKAS 125856, holotype). (**a**,**b**) Colonies on natural substrate. (**c**–**f**) Conidiophores. (**g**–**i**) Conidiogenous cells. (**j**) Germinating conidium. (**k**–**p**) Conidium. (**q**–**r**) Anverse and reverse colonies on PDA. Scale bars: (**c**–**f**) 50 μm, (**j**–**k**,**n**–**p**) 20 μm, and (**g**–**i**,**l**–**m**) 10 μm.

*Helicosporium multidentatum* X.J. Xiao, J. Ma & Y.Z. Lu, sp. nov., Figure 5.

Index Fungorum number: IF900460

Etymology: The epithet “*multidentatum*” refers to the multi-dentate integration protrusions conidiogenous cells.

Holotype: HKAS 125856

*Saprobic* on decaying wood in a terrestrial habitat. *Sexual morph*: Unknown. *Asexual morph*: Hyphomycetous, helicosporous. *Colonies* on the substratum superficial, effuse, gregarious, bright lime green. *Mycelium* partly immersed, partly superficial, composed of branched, septate, pale brown to brown hyphae. *Conidiophores* macronematous, mononematous, erect, flexuous or straight, unbranched, septate, apical sterile, cylindrical, 130–200 × 4–6 μm ($\bar{x}$ = 165 × 5 μm, n = 20), brown to dark brown, paler towards the apex, thick-walled, smooth-walled. *Conidiogenous cells* holoblastic, monoblastic to polyblastic, discrete, bladder-like, arising laterally from the lower portion of the conidiophores, hyaline to pale brown, smooth-walled. *Conidia* solitary, pleurogenous, helicoid, rounded at tip, 12–13 μm diam., and conidial filament 1.5–3 μm wide ($\bar{x}$ = 12.5 × 2.5 μm, n = 30), 105–130 μm long, tightly coiled 3^1^/_4_–3^3^/_4_ times, becoming loosely coiled in water, indistinctly multi-septate, guttulate, hyaline to pale green, smooth-walled.

Culture characteristics: Conidia germinated on water agar and produced germ tubes within 8 h. The colonies grew on PDA and had a circular shape with a flat surface and undulate edge. They reached a size of 35 mm in 5 weeks at 25 °C and exhibited a pale brown center with brown edges on PDA.

Material examined: CHINA, Guangxi Zhuang Autonomous Region, Guilin City, Qixingyan Scenic spot, on decaying wood, 4 May 2021, Xing-Juan Xiao & Jian Ma, QXY8 (HKAS 125856, holotype; GZAAS 22–2013, isotype), ex-type culture CGMCC, GZCC 22–2013. *Ibid*., QXY8-2 (HKAS 125855, paratype), living culture GZCC 23–0585.

Notes: Morphologically, *Helicosporium multidentatum* is similar to *H. hainanense* Y.Z. Lu & J.C. Kang and *H*. *vesicarium* Y.Z. Lu, J.C. Kang & K.D. Hyde in having brown to dark brown, unbranched and septate conidiophores with integrated multi-dentate protrusions arising laterally from its lower portion, and hyaline to pale green or yellowish, pleurogenous, helicoid conidia [10,11]. However, *H. multidentatum* differs from *H. hainanense* in having longer conidial filaments (105–130 μm vs. 55–60 μm), and from *H*. *vesicarium* in having smaller conidial diameter (12–13 μm vs. 13–18 μm) [10,11]. Phylogenetically, *H. multidentatum* forms a sister clade of *H*. *liuzhouense* with strong support and is distant from *H. hainanense* and *H*. *vesicarium* (Figure 2).

**Figure 6:**
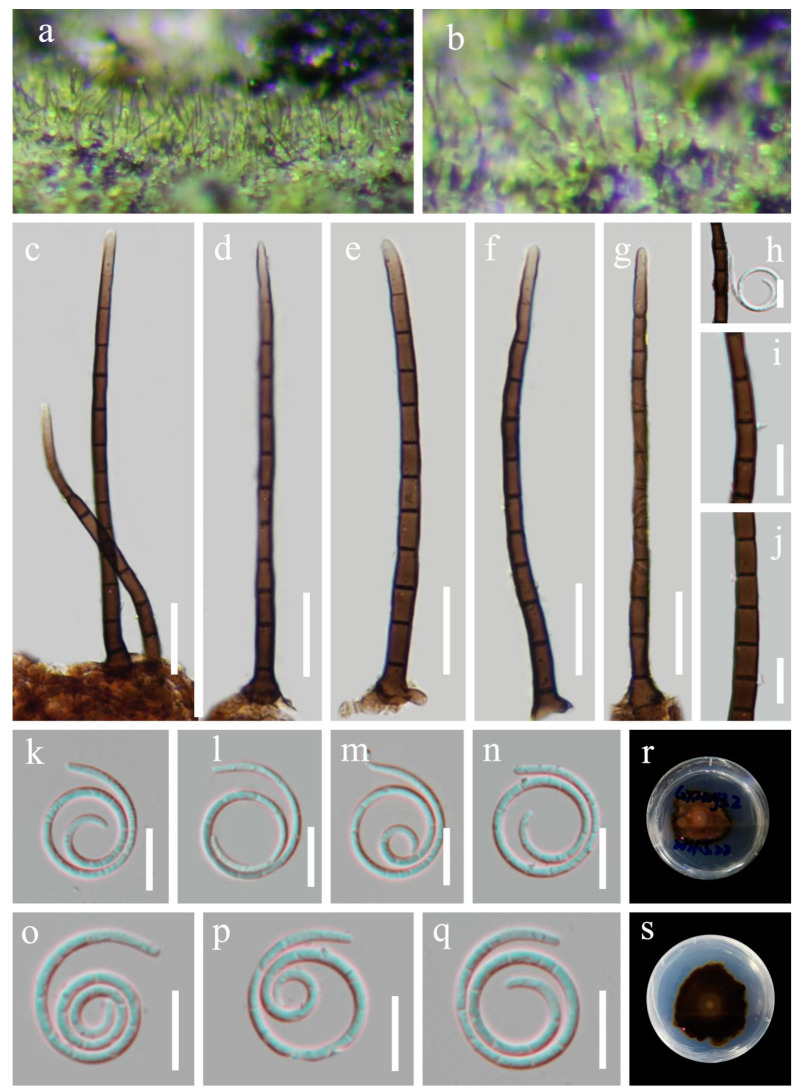
*Helicosporium nanningense* (HKAS 128858, holotype). (**a**,**b**) Colonies on natural substrate. (**c**–**g**) Conidiophores. (**h**–**j**) Conidiogenous cells. (**k**–**q**) Conidium. (**r**,**s**) Anverse and reverse colonies on PDA. Scale bars: (**c**–**g**) 20 μm and (**h**–**q**) 10 μm.

*Helicosporium nanningense* X.J. Xiao, J. Ma & Y.Z. Lu, sp. nov., Figure 6.

Index Fungorum number: IF900556

Etymology: The epithet “*nanningense*” named after the city in which the holotype was found.

Holotype: HKAS 128858

*Saprobic* on decaying wood in a terrestrial habitat. *Sexual morph*: Unknown. *Asexual morph*: Hyphomycetous, helicosporous. *Colonies* on the substratum superficial, effuse, gregarious, bright green. *Mycelium* partly immersed, partly superficial, branched, septate, pale brown to brown hyphae. *Conidiophores* macronematous, mononematous, flexuous or straight, unbranched, septate, apical sterile, cylindrical, 90–115 × 4–5 μm ($\bar{x}$ = 102 × 5 μm, n = 20), brown to dark brown, thick-walled, smooth-walled. *Conidiogenous cells* holoblastic, monoblastic to polyblastic, integrated, determinate, with denticles, arising laterally from the lower portion of the conidiophores as tiny tooth-like protrusions, hyaline to pale brown, smooth-walled. *Conidia* solitary, pleurogenous, helicoid, rounded at tip, 11–14 μm diam., and conidial filament 1.5–2.0 μm wide ($\bar{x}$ = 13 × 1.8 μm, n = 30), 82–92 μm long, tightly coiled 2–3^1^/_2_ times, becoming loosely coiled in water, indistinctly multi-septate, guttulate, hyaline to pale green, smooth-walled.

Culture characteristics: Conidia germinated on water agar and produced germ tubes within 12 h. The colonies grew on PDA and had a circular shape with a flat surface and undulate edge. They reached a size of 42 mm in 5 weeks at 25 °C and exhibited a brown center with nigger-brown edges on PDA.

Material examined: CHINA, Guangxi Medicinal Botanical Garden, Nanning City, on decaying wood in a terrestrial habitat, 4 May 2021, Xing-Juan Xiao & Jian Ma, GXZWY3.2 (HKAS 128858, holotype; GZAAS 23–0591, isotype), ex-type living culture CGMCC, GZCC 23–0587. *Ibid*., GXZWY3.5 (GZAAS 23–0592, paratype), living culture GZCC 23–0588.

Notes: Phylogenetically, *Helicosporium nanningense* shares a sister relationship to *H. multidentatum* with high statistical support (94% ML/1.00 PP). Morphologically, *H. nanningense* differs from *H. multidentatum* in having different conidiogenous cells (tiny tooth-like protrusions vs. integrated multi-dentate protrusions). Additionally, *H. nanningense* differs from *H. multidentatum* in having shorter conidiophores (90–115 µm vs. 130–200 µm) and larger conidia (105–130 µm vs. 82–92 µm). Moreover, *H. nanningense* is similar to *H. viridisporum* in conidiophores, conidiogenous cells, and conidial features [11], but the phylogenetic analysis result supports that they are distinct species.

## 6. Discussion

Helicosporous fungi are filamentous fungi whose conidia curve moves by at least 180 degrees in one plane or three-dimensional space as they grow longer [2,3,9,10,11,27,28,29,30]. Due to their ability to produce active secondary metabolites with distinct structures, these fungi have attracted scientists’ interest and become a popular study area. Numerous novel helicosporous taxa have recently been discovered in subtropical to tropical terrestrial and freshwater habitats [9,10,11,12,21,22,30,31,32]. In addition, a growing number of active secondary metabolites have been isolated from helicosporous fungi [33,34,35,36].

*Helicomyces* Link [37], *Helicosporium* [1], and *Helicoma* [38] are the three earliest described helicosporous genera. Based on morphological characteristics, researchers including Linder [2], Moore [39], and Goos [3,27,28,29] carried out systematic classification studies on these three genera. Tsui et al. [40] conducted a phylogenetic analysis of helicosporous fungi. They discovered that the species of *Helicomyces*, *Helicosporium*, and *Helicoma* did not cluster within their respective genus-level taxonomy units but instead interbred and dispersed within the family Tubeufiaceae. Kuo and Goh [41] also reported the chaotic phylogenetic relationships between these three genera. Lu et al. [10] reevaluated these three genera, redefined their generic concepts based on morphological and phylogenetic evidence, and provided recommendations for classifying and identifying helicosporous fungi. However, some taxa within these genera still require additional morphological and molecular data to resolve their taxonomic issues. For example, Boonmee et al. [7] combined *Helicosporium* sp. NBRC 9014 (as *Tubeufia cerea* NBRC 9014 in Tsui et al. [40]) with *H. vegetum* based on phylogenetic analyses. However, Lu et al. [10] disagreed with this treatment as *Helicosporium* sp. NBRC 9014 did not cluster with other *H. vegetum* strains in the multi-gene phylogenetic tree. The taxonomic status of this strain remains unresolved due to insufficient morphological information [10].

Lu et al. [11] highlighted the challenge of taxonomic studies on helicosporous fungi due to their similar morphological characteristics. In this study, three new helicosporous fungi, namely, *Helicosporium liuzhouense*, *H. multidentatum,* and *H. nanningense*, were identified using morphological and phylogenetic analyses, with supporting evidence from the PHI test. *Helicosporium liuzhouense* and *H. nanningense* share similarities with *H. sexuale* and *H. viridisporum* in terms of conidiophores, conidiogenous cells, and conidial features, respectively, while *H. multidentatum* is comparable to *H. hainanense* and *H*. *vesicarium*. Notably, they have distinct phylogenetic positions (Figure 2). These findings reinforce the significance of molecular data in precisely distinguishing helicosporous hyphomycetes.

A checklist of accepted *Helicosporium* species is provided in this study (Table 2). Nine species are found in freshwater habitats and 11 species in terrestrial habitats, including the newly described species in this study. *Helicosporium sexuale* occurs in both freshwater and terrestrial habitats. Among them, 18 species are reported only in their helicosporous asexual morph, while three species, *viz., H. flavum*, *H. sexuale,* and *H. vegetum,* have asexual–sexual links that have been confirmed by molecular data. The taxonomic status of 15 species has been determined through phylogenetic analyses, while six species do not have any molecular data and require further research to determine their phylogenetic relationships [42,43,44,45]. A key to the species accepted in *Helicosporium* is provided as well.

Key to species of *Helicosporium*1. Fresh colonies on decaying woody substrate are bright green····················································································································21. Fresh colonies on decaying woody substrate are hyaline, gray, yellow, yellow green, or dark brown··························52. Conidiophores unbranched, with multi-dentate protrusions conidiogenous cells····················································*H. multidentatum*2. Conidiophores unbranched, with tiny tooth-like protrusions conidiogenous cells··············································································33. Conidiophores < 120 μm long··········································································*H. nanningense*3. Conidiophores > 120 μm long····················································44. Conidiophores (102) 110–180 (213) µm long, 4–5 µm wide, conidia 13–15 µm diam., 90–105 µm long····················································*H. liuzhouense*4. Conidiophores 80–206 µm long, 3–7 µm wide, conidia 12–14 µm diam., 75–97 µm long····················································*H. viridisporum* or *H. viridisporum*5. Fresh colonies on decaying woody substrate are yellow green····················································65. Fresh colonies on decaying woody substrate are hyaline, gray, yellow, or dark brown····················································146. Conidiophores with bladder-like protrusions conidiogenous cells····················································76. Conidiophores with tiny tooth-like protrusions conidiogenous cells··························87. Conidiophores < 120 μm long··········································································*H. vesicarium*7. Conidiophores > 120 μm long··········································································*H. hainanense*8. Conidia are acropleurogenous····················································98. Conidia are pleurogenous and apical sterile in conidiophores··························109. Conidiophores 450–550 µm long··························*H. thailandense*9. Conidiophores 184–257 µm long····················································*H. flavidum*10. Conidiophores < 180 μm long····················································1110. Conidiophores > 180 μm long····················································1311. Conidiophores unbranched, conidia 10–14 µm diam., 70–90 µm long····················································*H. aquaticum*11. Conidiophores rarely branched····················································1212. Conidiophores 125–320 µm long, conidia 13–21 µm diam., 100–130 µm long··························*H. setiferum*12. Conidiophores 60–129 µm long, conidia 11–20 µm diam., 68–91 µm long····················································*H. sexuale*13. Conidiophores 30–360 µm long, conidia 10–15 μm diam.··························*H. vegetum*13. Conidiophores 250–425 µm long, conidia 20–23 μm diam.··························*H. viridiflavum*14. Fresh colonies on decaying woody substrate are yellow··························1514. Fresh colonies on decaying woody substrate are hyaline, gray, or dark brown··························1715. Conidia are acropleurogenous, conidiophores 36–48 µm long··························*H. flavum*15. Conidia are pleurogenous and apical sterile in conidiophores··························1616. Conidiophores with bladder-like protrusions conidiogenous cells··························*H. flavisporum*16. Conidiophores with tiny tooth-like protrusions conidiogenous cells··························*H. luteosporum*17. Fresh colonies on decaying woody substrate are hyaline, branched conidiophores 200–300 µm long·································*H. albidum*17. Fresh colonies on decaying woody substrate are gray, or dark brown··························1818. Fresh colonies on decaying woody substrate are gray, conidiophores 350–400 µm long, with bladder-like protrusions conidiogenous cells··················*H. neesii*18. Fresh colonies on decaying woody substrate are dark brown····················································1919. Conidiophores are unbranched with tooth-like protrusions conidiogenous cells····················································*H. murinum*19. Conidiophores are rarely branched··············································································2020. Tooth- or bladder-like protrusions conidiogenous cells, conidia 6–9 µm diam.····················································*H. decumbens*20. Tooth-like protrusions conidiogenous cells, conidia 11.4–19 µm diam.··························*H. melghatianum*

## Figures and Tables

**Table 1:** List of taxa used in this study along with their corresponding GenBank accession for DNA sequences.

Taxon	Strain	GenBank Accessions			
		ITS	LSU	*tef1α*	*rpb2*
*Acanthostigma chiangmaiense*	MFLUCC 10-0125^T^	JN865209	JN865197	KF301560	-
*Acanthostigma patagonicum*	BBB MVB 573	JN127358	JN127359	-	-
*Acanthostigma perpusillum*	UAMH 7237^T^	AY916492	AY856892	-	-
*Helicoma vaccinii*	CBS 216.90	AY916486	AY856879	-	-
*Helicosporium aquaticum*	MFLUCC 17-2008^T^	MH558733	MH558859	MH550924	MH551049
*Helicosporium flavisporum*	MFLUCC 17-2020^T^	MH558734	MH558860	MH550925	MH551050
*Helicosporium flavum*	MFLUCC 16-1230^T^	KY873626	KY873621	KY873285	-
*Helicosporium hainanense*	GZCC 22-2006^T^	OP508730	OP508770	OP698081	OP698070
***Helicosporium liuzhouense***	**GZCC 22-2014^T^**	**OQ981394**	**OQ981402**	**OQ980476**	**OQ980474**
***Helicosporium liuzhouense***	**GZCC 23-0586**	**OR066416**	**OR066423**	**OR058862**	**OR058855**
*Helicosporium luteosporum*	MFLUCC 16-0226^T^	KY321324	KY321327	KY792601	-
*Helicosporium luteosporum*	MFLUCC 16-1233	-	KY873624	-	-
***Helicosporium multidentatum***	**GZCC 22-2013^T^**	**OQ981395**	**OQ981403**	**OQ980477**	**OQ980475**
***Helicosporium multidentatum***	**GZCC 23-0585**	**OR066417**	**OR066424**	**OR058863**	**OR058856**
***Helicosporium nanningense***	**GZCC 22-2175^T^**	**OR066418**	**OR066425**	**OR058864**	**OR058857**
***Helicosporium nanningense***	**GZCC 23-0588**	**OR066419**	**OR066426**	**OR058865**	**OR058858**
*Helicosporium setiferum*	BCC 3332	AY916490	AY856907	-	-
*Helicosporium setiferum*	BCC 8125	AY916491	-	-	-
*Helicosporium setiferum*	MFLUCC 17-1994^T^	MH558735	MH558861	MH550926	MH551051
*Helicosporium setiferum*	MFLUCC 17-2006	MH558736	MH558862	MH550927	MH551052
*Helicosporium setiferum*	MFLUCC 17-2007	MH558737	MH558863	MH550928	MH551053
*Helicosporium sexuale*	GZCC 22-2007	OP508731	OP508771	OP698082	OP698071
*Helicosporium sexuale*	MFLUCC 16-1244^T^	MZ538503	MZ538537	MZ567082	MZ567111
*Helicosporium* sp.	NBRC 9014	AY916489	AY856903	-	-
*Helicosporium thailandense*	MFLUCC 18-1407^T^	MT627698	MN913718	MT954371	-
*Helicosporium vegetum*	CBS 254.75	-	DQ470982	DQ471105	-
*Helicosporium vegetum*	CBS 269.52	AY916487	AY856893	-	-
*Helicosporium vegetum*	CBS 941.72^T^	AY916488	AY856883	-	-
*Helicosporium vegetum*	NBRC 30345	-	AY856896	-	-
*Helicosporium vesicarium*	MFLUCC 17-1795^T^	MH558739	MH558864	MH550930	MH551055
*Helicosporium viridiflavum*	MFLUCC 17-2336^T^	MH558738	-	MH550929	MH551054
*Helicosporium viridisporum*	GZCC 22-2008^T^	OP508736	OP508776	OP698087	OP698076

Note: “T” represents the ex-type strain. Newly generated sequences are represented in bold. “-” indicates that no sequence data are available in GenBank.

**Table 2:** Checklist of accepted *Helicosporium* species.

Species	Habitats	Distribution	Molecular Data	References
*H. albidum*	Terrestrial	Belgium, Britain (Birminghan)	No	Grove 1886 [43]
*H. aquaticum*	Freshwater	Thailand	Yes	Lu et al. 2018 [10]
*H. decumbens*	Terrestrial	Austria, Brazil	No	Linder 1929 [2]
*H. flavidum*	Freshwater	China	No	Hsieh 2021 [44]
*H. flavisporum*	Freshwater	Thailand	Yes	Lu et al. 2018 [10]
*H. flavum*	Freshwater	Thailand	Yes	Brahmanage et al. 2017 [14]
*H. hainanense*	Terrestrial	China	Yes	Lu et al. 2022 [11]
*H. liuzhouense*	Freshwater	China	Yes	This study
*H. luteosporum*	Terrestrial	Thailand	Yes	Lu et al. 2017 [9]
*H. melghatianum*	Terrestrial	India	No	Dharkar et al. 2010 [42]
*H. murinum*	Terrestrial	Argentina, Austria, Brazil, Canada, China, Cuba, Malaysia, USA	No	Linder 1929 [2]; Goos 1989 [3]; Zhao et al. 2007 [6]
*H. multidentatum*	Terrestrial	China	Yes	This study
*H. nanningense*	Terrestrial	China	Yes	This study
*H. neesii*	Terrestrial	USA	No	Moore 1957 [39]
*H. setiferum*	Freshwater	Thailand	Yes	Lu et al. 2018 [10]
*H. sexuale*	Freshwater, Terrestrial	China, Thailand	Yes	Boonmee et al. 2021 [8]; Lu et al. 2022 [11]
*H*. *thailandense*	Freshwater	Thailand	Yes	Dong et al. 2020 [12]
*H. vegetum*	Terrestrial	Worldwide	Yes	Boonmee et al. 2014 [7]
*H. vesicarium*	Freshwater	Thailand	Yes	Lu et al. 2018 [10]
*H. viridiflavum*	Terrestrial	Thailand	Yes	Lu et al. 2018 [10]
*H. viridisporum*	Freshwater	China	Yes	Lu et al. 2022 [11]

## Data Availability

All sequences generated in this study were submitted to GenBank database.
